# Monitoring the progress towards the elimination of hepatitis B and C in Sweden: estimation of core indicators for 2015 and 2018

**DOI:** 10.1186/s12879-022-07886-2

**Published:** 2022-11-25

**Authors:** Soledad Colombe, Maria Axelsson, Soo Aleman, Ann-Sofi Duberg, Josefine Lundberg Ederth, Viktor Dahl

**Affiliations:** 1 Public Health Agency of Sweden, Solna, Sweden; 2 European Programme for Intervention Epidemiology Training (EPIET), European Centre for Disease Prevention and Control (ECDC), Solna, Sweden; 3 Outbreak Research Team, Department of Public Health, Institute of Tropical Medicine, Antwerp, Belgium; 4 Department of Infectious Diseases, Karolinska University Hospital/Karolinska Institutet, Stockholm, Sweden; 5 Department of Infectious Diseases, School of Medical Sciences, Faculty of Medicine and Health, Örebro University, Örebro, Sweden

**Keywords:** Disease elimination, Viral hepatitis C, Viral hepatitis B, Surveillance

## Abstract

**Introduction:**

To monitor Sweden’s progress towards the WHO goal of eliminating viral hepatitis, we estimated the prevalence, notification rate, and liver-related morbidity and mortality for diagnosed hepatitis B virus (HBV) and hepatitis C virus (HCV) infections in 2015 and 2018.

**Methods:**

We identified cases of hepatitis B and C within the National System for Notifiable Diseases and obtained data on treatment and whether the case was deceased or not. We calculated prevalence, notification rates per 100,000, and proportion of newly diagnosed cases of hepatitis with liver disease at the time of diagnosis, and proportion of all deceased cases who died from liver disease. We calculated Poisson 95% confidence intervals (CIs) around the notification rates and Wilson 95% CIs around prevalence and mortality estimates.

**Results:**

In 2015 and 2018, the prevalence of diagnosed HBV infections was 0.20% [95% CI: 0.19–0.20] and 0.21% [0.20–0.21]. Notification rates per 100,000 for HBV infections were 13.02 [12.32–13.76] and 7.71 [7.18–8.27]. HBV liver-related morbidity was 2.65% [1.90–3.68] and 2.16% [1.35–3.43]. HBV liver-related mortality was 20.00% [14.81–26.44] and 17.95% [13.20–23.94]. In 2015 and 2018, the prevalence of diagnosed HCV-infections was 0.24% [0.24–0.25] and 0.18% [0.18–0.19]. Notification rates per 100,000 for HCV infections were 15.92 [15.14–16.73] and 13.05 [12.36–13.77]. HCV liver–related morbidity was 8.14% [6.89–9.60] and 3.90% [2.99–5.08]. HCV liver–related mortality was 27.08% [24.54–29.77] and 26.90% [24.12–29.88].

**Conclusions:**

All indicators decreased or remained stable between 2015 and 2018, indicating progress in the elimination of viral hepatitis, especially for HCV infection.

## Introduction

In 2016, the World Health Organization (WHO) set a goal to eliminate viral hepatitis as a major public health threat by 2030, defined as a reduction in hepatitis-related deaths by 65% and new chronic HBV and HCV infections by 90%, using 2015 as the baseline year [1]. This strategy was accompanied by a framework to help monitor and evaluate the progress by following 10 core indicators (including incidence, prevalence, and mortality) [2]. The European Centre for Disease Control (ECDC) also created its own framework and set of indicators specific for the EU, which additionally includes morbidity at time of diagnosis [3].

In Sweden, all cases of hepatitis B and C are notifiable by law, and on average 1500–2000 new notifications are recorded annually for both HBV and HCV infection [4, 5]. Modelling studies from 2015–2016 estimated that, in Sweden, between 10,000 and 68,500 [6, 7] individuals were chronically infected with HBV and between 35,000 and 45,000 with HCV [8, 9].

The majority of HBV infections diagnosed in Sweden are in immigrants from high-endemic countries who likely were infected at birth or during childhood, before they arrived in Sweden. Among domestic infections, the most common modes of transmission are heterosexual contact and drug injection [4]. HBV vaccination is offered in Sweden for free for children since 2016, which led to a rise in coverage from 53 to 92% among 2-year-olds between 2015 and 2018 [10]. Pregnant women are tested for HBV in the national screening program for pregnant women, and all children to HBV-positive mothers are vaccinated at birth. In addition, highly viremic pregnant women receive antiviral treatment, and their babies receive anti-HBV immunoglobulins at birth. The most recent guidelines from 2019 recommend suppressive antiviral treatment for patients with chronic hepatitis B and progressive liver disease, which is given free of charge [11].

In contrast to HBV, most HCV infections have been acquired within Sweden, with the most common mode of transmission being injection drug use [5]. A study from 2018 reported viremic HCV infection among 56% of people who participated in the needle-exchange program in Stockholm [12]. Until 2017, few sites were providing needle-exchange programs in Sweden, but this is quickly changing with 17 out of 21 regions providing this service as of 2020. Since 2014, a combination of direct-acting antivirals (DAAs) has been available in Sweden for treatment of HCV infection. DAAs have fewer and milder side effects than previous treatment regimens, and have cure rates above 95% after treatment for 8–12 weeks [13]. Due to high costs, they were initially only recommended and subsidised for those with advanced liver fibrosis, but since January 2018 DAAs are recommended and free of charge for all Swedish residents with hepatitis C irrespective of the fibrosis stage [13].

To monitor Sweden’s progress towards the elimination of viral hepatitis, we developed a plan for follow-up of a selected set of indicators (i.e., prevalence and notification rate of diagnosed HBV and HCV infections, liver-related morbidity at time of diagnosis, and liver-related mortality) suggested by the WHO and ECDC and adapted for the Swedish context so that they could be calculated by data available from our routine surveillance for viral hepatitis and from other national databases already in place. We then calculated these indicators for 2015 (the baseline year, as suggested by the WHO) and for 2018, as the most recent data available at time of the first follow-up.

## Methods

### Study design and data collection

We conducted a retrospective observational study with the year 2015 as the baseline and the year 2018 as the first year of follow-up.

**Fig. 1:**
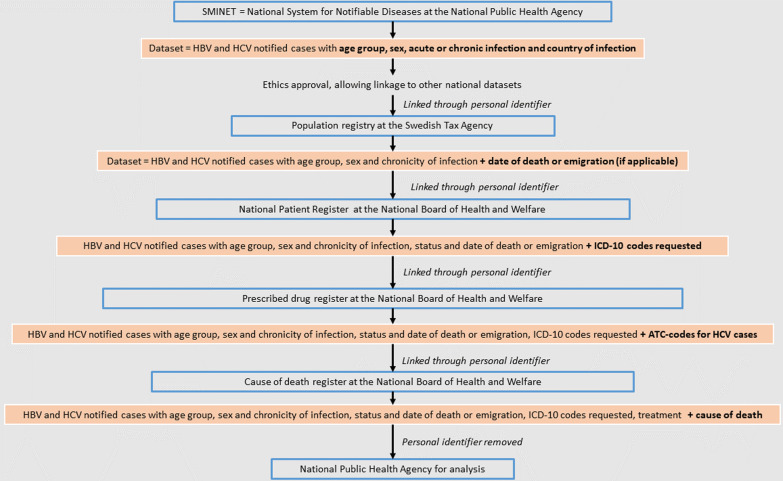
Flow of database linkage between the different national authorities in Sweden

We identified all cases of hepatitis B and C reported to the National System for Notifiable Diseases (SmiNet) and with a permanent personal identification number (see definition below) from the years 1969 and 1990 (the time-points when hepatitis B and C became notifiable by law, respectively [4, 5]) until 31 December 2018 (Fig. 1). In Sweden, both hepatitis B and C are notifiable by law, both by the clinicians and by the laboratories [14]. The two notifications are then merged into a “case” in the national database for notifiable infections “SmiNet”. The clinical notification should include, among other clinical data, information about acute or chronic infection, plausible route of transmission as well as the country of infection. Though not mandatory, the laboratory method (serology or PCR) can be reported as well as if the result was positive, negative or indeterminate. Subsequent tests are reported under the same personal identification number but are not automatically performed. We extracted the personal identification number, age at time of notification, sex, country of infection, date of notification in SmiNet, and chronicity of infection as reported by the clinician and laboratory method used for diagnosis (serology or PCR). Cases were linked by personal identification number to the population registry at the Swedish Tax Agency to add date of death, emigration, or removal from the population registry for other reasons [15].

These data were further linked to the national registers at the National Board of Health and Welfare, the National Patient Register, the Prescribed Drug Register, and the Cause of Death Register (Fig. 1). In the Patient Register all inpatient hospital visits and all outpatient visits to specialist care are recorded with diagnoses coded according to the 10^th^ International Classification of Diseases (ICD-10) [16]. We used the ICD-10 codes suggested in the WHO framework [2]. The following specific codes were extracted for diagnoses of diseases attributable to HBV and HCV infections: HCC or liver cancer of unknown type (C22.0 and C22.9), liver cirrhosis (K74.4- K74.6), and chronic liver disease (K72, K73, K74, K75). In the Prescribed Drug Register all prescribed drugs have been recorded on an individual level since July 2005 [17], and we extracted treatment for HCV infection using the following Anatomical Therapeutic Chemical Classification System (ATC) codes: L03AB for IFNs (Interferons—IFNs and pegylated (peg)-IFNs) and J05AP for DAAs and ribavirin. Finally, we linked cases to the Cause of Death Register [18]. We specifically extracted ICD-10 codes for liver disease as defined above, and causes of death with these ICD-10 codes will be further referred to as “liver-related deaths” in this study. Aggregated data on size and demographics of the general population were extracted from Statistics Sweden [19].

### Operational definitions and assumptions

#### Personal identification number

The personal identification number is a unique national official identifier given to each Swedish resident and follows a person for their entire life. It is obtained at birth or when immigrating to Sweden. Because all national registers use this unique number, it is possible to link different data sources. After arriving in Sweden, it usually takes at least 3 months to be registered and obtain the permanent personal identification number for migrants. In the meantime temporary registration numbers are generated at the local level when visiting health care facilities, and one person could get more than one temporary number. These cannot be linked across registers. Once a person obtains a permanent personal identification number, it is not possible to link data associated with the temporary registration number to the permanent personal identification number. We refer to this temporary registration number as the “temporary personal identification number” in this study. In this group we also included the notifications where the full permanent personal identification number was missing for unknown reason. We assumed cases reported with a temporary personal identification number would be reported again under a permanent personal identification number if they remained in Sweden. We therefore focused our analyses on those with permanent personal identification number to avoid duplicate notifications and to be able to link to other national registers.

#### Clearance and cure rates

We estimated clearance and cure rates in order to calculate the prevalence of chronically infected individuals with HBV or HCV diagnosis. The percentage of spontaneous clearance after notification for acute HBV infections was estimated to be 5% for infants (< 1 year of age) and 95% for the remaining cases [2]. We assumed no spontaneous clearance when the clinician notified the infection as a “chronic” HBV infection.

In Sweden, HCV infections used to be reported based on a positive anti-HCV IgG serology test according to the case definition used until 2020. Thus, we could not know if these infections were chronic or had cleared spontaneously. Based on a meta-analysis [20], we assumed a 37% spontaneous clearance rate for all reported and non-treated HCV infections. But estimations of spontaneous clearance vary. The WHO estimates it to be 15% -45%, the ECDC 20%, United States Centers for Disease Control and Prevention 50% and The Swedish Reference Group for Antiviral Therapy estimates it to be 25%–50%. Due to this variation in the estimates, a sensitivity analysis around the prevalence was conducted assuming a spontaneous clearance rate for all reported and non-treated HCV infections of 25% and 50%.

For treated HBV-infected individuals, the functional cure rate is low [19], and for the analyses we therefore assumed no cure rates after antiviral treatment [11], and thus did not collect medication data. For treated HCV-infected individuals, we assumed treatment cure rates of 50% for those who had received IFNs in combination with ribavirin [21] and 95% for those who had received DAAs [13]. For the analyses, we assumed that there were no re-infections after HCV-treatment.

These clearance and cure rates were applied to the number of cases reported in SmiNet as described in the *Data Analysis* paragraph.

#### Diagnosis of liver disease

Liver disease at the time of HBV or HCV diagnosis was defined as the occurrence of one of the codes specified by the WHO (see above) in the Patient Register within one year before or after the date of HBV/HCV notification, for those notified in 2015 and 2018.

### Data analysis

For continuous data we calculated the median and interquartile range. We followed the WHO and ECDC guidelines for calculating the prevalence, notification rate, liver-related morbidity, and liver-related mortality [2, 3].

The prevalence of diagnosed chronic HBV and HCV infections in 2015 and in 2018 was calculated by dividing the total number of individuals living with a diagnosis of chronic HBV or HCV infection by the total population in Sweden in the respective years. In 2015 and 2018, 9,851,017 and 10,230,185 people were residing in Sweden, respectively. The numerator included the total number of cases reported in SmiNet since HBV and HCV infections became notifiable diseases, excluding cases where deaths and emigrations had occurred as of January 1st of the next year (2016 and 2019), and the estimated number of cleared infections as described above (spontaneously or by treatment).

For both hepatitis types, we calculated the notification rates for 2015 and 2018, respectively, as the number of new cases of hepatitis reported in SmiNet in each year divided by the total number of residents in Sweden in that year.

For both prevalence and notification rates, we made additional calculations also including those with a temporary personal identification number.

We calculated the rates of liver-related morbidity at the time of HBV or HCV diagnosis for each year (2015 and 2018) as the proportion of newly reported cases who also had received at least one of the ICD-10 codes indicating liver disease (as specified by the WHO) within one year before or after the date of diagnosis of the infection.

We calculated liver-related mortality attributable to HBV and HCV as the number of deaths with at least one ICD-10 code indicating liver disease (as specified by the WHO) as the cause of death, divided by the total number of deaths among people living with HBV or HCV infection for 2015 and 2018, respectively.

We calculated Wilson 95% confidence intervals (CIs) for proportions and Poisson 95% CIs for notification rates. P-values for the differences in proportions were calculated using Fisher’s exact test. All analyses were conducted in STATA 15.1.

## Results

### Hepatitis B

**Fig. 2:**
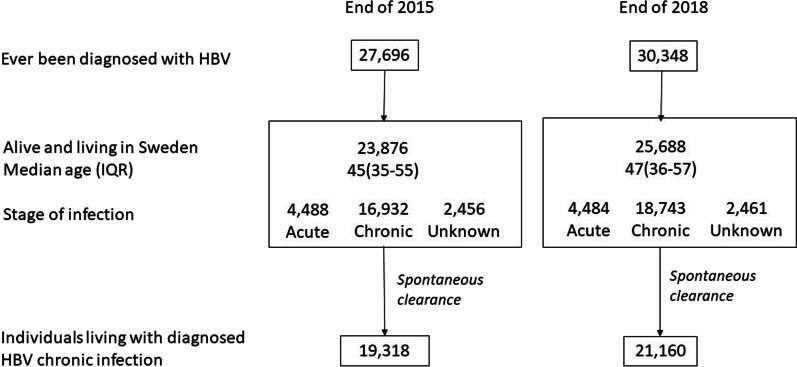
Flow of cases from notifications to resulting prevalence in 2015 and 2018 for HBV-diagnosed infections among individuals with permanent personal identification number

The flow of notifications and applied assumptions is described in Fig. 2. The total numbers of individuals with a diagnosed chronic HBV infection were 19,318 and 21,160 by the end of 2015 and 2018, respectively. This corresponds to a prevalence of 0.20% [95% CI: 0.19–0.20] by the end of 2015 and 0.21% [95% CI: 0.20–0.21] by the end of 2018 (Table 1).

**Table 1:** Prevalence of diagnosed chronic hepatitis B virus (HBV) infection in Sweden, by the end of 2015 and 2018, by sex

	2015			2018		
	Number of HBV infections	General population	Prevalence* (%) [95% CI]	Number of HBV infections	General population	Prevalence* (%) [95% CI]
Sex						
Women	8489	4,920,051	0.17 [0.17–0.18]	9341	5,087,747	0.18 [0.18–0.19]
Men	10,803	4,930,966	0.22 [0.215–0.223]	11,790	5,142,438	0.23 [0.225–0.234]
Unknown	26			29		
Total	19,318	9,851,017	0.20 [0.19–0.20]	21,160	10,230,185	0.21[0.20–0.21]

*Among those with a permanent personal identification number. *CI* confidence interval

In 2015 and 2018, notification rates of HBV were 13.02 [95% CI: 12.32–13.76] per 100,000 persons and 7.71 [95% CI: 7.18–8.27] per 100,000 persons, respectively (Table 2).

**Table 2:** Notification rate of hepatitis B virus (HBV) infection per 100,000 in Sweden in 2015 and 2018, by age and sex

	2015			2018		
Variable	Number of new hepatitis B notifications	General population	Notification rate per 100,000 persons* [95%CI]	Number of new hepatitis B notifications	General population	Notification rate per 100,000 persons * [95%CI]
Sex						
Women	433	4,920,051	8.80 [7.99–9.67]	346	5,087,747	6.80 [6.10–7.56]
Men	850	4,930,966	17.24 [16.10–18.44]	443	5,142,438	8.61 [7.83–9.46]
Age at diagnosis (years)						
Infant	0	115,879	0.00 [0.00–3.18]	2	116,840	1.71 [0.21–6.18]
1–9	11	1,059,458	1.04 [0.52–1.86]	11	1,107,132	0.99 [0.50–1.78]
10–19	150	1,064,279	14.09 [11.93–16.54]	67	1,155,271	5.80 [4.49–7.37]
20–29	292	1,338,861	21.81 [19.38–24.46]	136	1,338,915	10.16 [8.52–12.02]
30–39	379	1,227,641	30.87 [27.84–34.14]	241	1,330,260	18.12 [15.90–20.55]
40–49	241	1,314,367	18.34 [16.09–20.80]	177	1,294,175	13.68 [11.74–15.85]
50 +	210	3,730,533	5.63 [4.89–6.44]	155	3,887,593	3.99 [3.38–4.67]
Country of infection						
Sweden	127	9,851,017	1.29 [1.08–1.53]	54	10,230,185	0.53 [0.40–0.69]
Other	1024	9,851,017	10.39 [9.77–11.05]	631	10,230,185	6.17 [5.70–6.67]
Un-known	132			104		
Total	1283	9,851,017	13.02 [12.32–13.76]	789	10,230,185	7.71 [7.18–8.27]

*Among those with a permanent personal identification number. *CI* confidence interval

Most HBV infections were reported as chronic, including 87% (1111/1283) in 2015 and 92% (726/789) in 2018. In 2015, the estimated liver-related morbidity at the time of diagnosis was 2.65% (34/1,283) [95% CI: 1.90–3.68] while it was 2.16% (17/789) [95% CI: 1.35–3.43] in 2018.

Among the 180 HBV-infected individuals who died in 2015, 36 (20% [95% CI: 14.81–26.44]) died of liver disease. In 2018, 195 HBV-infected individuals died of which 35 (17.95% [95% CI: 13.20–23.94]) died of liver disease. The proportion of deaths attributable to HBV infection was similar between men and women in both years (p > 0.05). There were no liver-related deaths among HBV-infected individuals younger than 30 years of age. We did not find a statistically significant association between age and liver-related HBV deaths in our data.When adding notifications with a temporary personal identification number, the total numbers of notifications of diagnosed chronic HBV infections were 35,592 and 39,107 for 2015 and 2018, respectively, corresponding to a prevalence of 0.36% [95% CI: 0.36–0.37] by the end of 2015 and 0.38% [95% CI: 0.38–0.39] by the end of 2018.

When including notifications with a temporary personal identification number, the total numbers of new HBV notifications were 2376 and 1128, for 2015 and 2018, respectively, corresponding to notification rates of 24.12 [95% CI: 23.16–25.11] per 100,000 and 11.03 [95% CI: 10.39–11.69] per 100,000, respectively.

### Hepatitis C

**Fig. 3:**
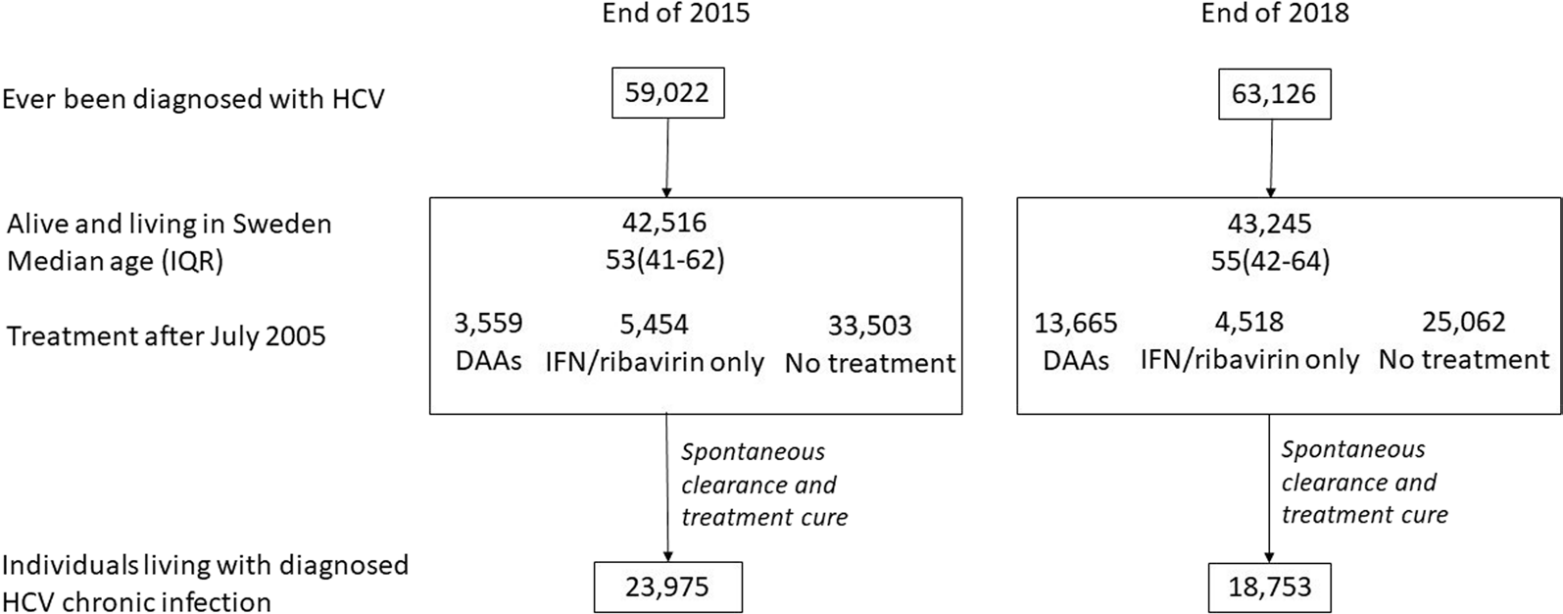
Flow of cases from notifications to resulting prevalence in 2015 and 2018 for HCV-diagnosed infections among individuals with permanent personal identification number

The flow of notifications and applied assumptions is described in Fig. 3. After assumptions of spontaneous clearance and treatment cure, there were an estimated 23,975 individuals with diagnosed chronic HCV infections in 2015 and 18,753 in 2018. The prevalence of diagnosed HCV infections was then 0.24% [95% CI: 0.24–0.25] by the end of 2015 and 0.18% [95% CI: 0.18–0.19] by the end of 2018 (Table 3).

**Table 3:** Prevalence of diagnosed chronic hepatitis C virus (HCV) infections in Sweden by the end of 2015 and 2018, by sex

Variable	2015			2018		
	Number of HCV notifications	General population	Prevalence*(%) [95%CI]	Number of HCV notification	General population	Prevalence* (%) [95%CI]
Sex						
Women	8549	4,920,051	0.17 [0.17–0.18]	6812	5,087,747	0.13 [0.13–0.14]
Men	15,421	4,930,966	0.31 [0.31–0.32]	11,939	5,142,438	0.23 [0.23–0.24]
Unknown	5			2		
Total	23,975	9,851,017	0.24 [0.24–0.25]	18,753	10,230,185	0.18 [0.18–0.19]

*Among those with a permanent personal identification number. *CI* confidence interval

The sensitivity analyses for a clearance rate of 25% and 50% led to a prevalence of 0.28% [95% CI: 0.28–0.29] and 0.20% [95% CI: 0.20–0.20], respectively, in 2015, and 0.21% [95%CI: 0.21–0.22] and 0.15% [95%CI: 0.15–0.15], respectively, in 2018.

In 2015 and 2018, notification rates of newly diagnosed HCV infections were 1568 infections or 15.92 [95% CI: 15.14–16.73] per 100,000 persons in 2015 and 1335 infections or 13.05 [95% CI: 12.36–13.77] per 100,000 persons, respectively (Table 4).

**Table 4:** Notification rate of hepatitis C virus (HCV) infections per 100,000 in Sweden in 2015 and 2018, by age and sex

	2015			2018		
	Number of new hepatitis C notifications	General population	Notification rate per 100,000 persons* [95%CI]	Number of new hepatitis C notifications	General population	Notification rate per 100,000 persons* [95%CI]
Sex						
Women	540	4,920,051	10.98 [10.07–11.94]	474	5,087,747	9.32 [8.50–10.19]
Men	1028	4,930,966	20.85 [19.59–22.16]	861	5,142,438	16.74 [15.64–17.90]
Age at first dia-gnosis (years)						
Infant	1	115,879	0.86 [0.02–4.81]	1	116,840	0.86 [0.02–4.77]
1–9	14	1,059,458	1.32 [0.72–2.22]	9	1,107,132	0.81 [0.37–1.54]
10–19	207	1,064,279	19.45 [16.89–22.29]	155	1,155,271	13.42 [11.39–15.70]
20–29	426	1,338,861	31.82 [28.87–34.99]	387	1,338,915	28.90 [26.10–31.93]
30–39	279	1,227,641	22.73 [20.14–25.56]	243	1,330,260	18.27 [16.04–20.71]
40–49	103	1,314,367	7.84 [6.40–9.50]	99	1,294,175	7.65 [6.22–9.31]
50 +	539	3,730,533	14.45 [13.25–15.72]	442	3,887,593	11.37 [10.33–12.48]
Country of infection						
Sweden	1017	9,851,017	10.32 [9.70–10.98]	871	10,230,185	8.51 [7.96–9.10]
Other	212	9,851,017	2.15 [1.87–2.46]	193	10,230,185	1.89 [1.63–2.17]
Un-known	339			271		
Total	1568	9,851,017	15.92 [15.14–16.73]	1335	10,230,185	13.05 [12.36–13.77]

*Among those with a permanent personal identification number. *CI* confidence interval

Among the 1568 new notifications of HCV reported in 2015, 127 were diagnosed with at least one diagnosis of liver disease at the time of HCV diagnosis, and the liver-related morbidity at the time of diagnosis was 8.14% [95% CI: 6.89–9.60]. Liver-related morbidity was 3.90% (52/1335) [95% CI: 2.99–5.08] in 2018.

Among the 1108 HCV-infected individuals who died in 2015, 300 (27.08% [95% CI: 24.54–29.77]) died of liver disease. Among the 907 HCV-infected individuals who died in 2018, 244 (26.90% [95% CI: 24.12–29.88]) died of liver disease. The proportion of deaths attributable to HCV infection was similar between men and women in both years (p > 0.052). There were no deaths attributable to HCV infection in those younger than 20 years of age. The proportion of deaths was overall significantly different and higher in the older age groups (data not shown).

When adding notifications with a temporary personal identification number to our analysis, the total numbers of diagnosed chronic HCV infections were 27,130 in 2015 (prevalence 0.28% [95% CI: 0.27–0.28]) and 22,424 in 2018 (prevalence 0.22% [95% CI: 0.22–0.22]).

When including notifications with a temporary personal identification number, the total number reported in 2015 was 1959 infections or 18.90 [95% CI: 18.07–19.76] per 100,000 persons and in 2018 there was 1609 infections or 15.13 [95% CI: 14.40–15.89] per 100,000 persons.

## Discussion

This is the Public Health Agency of Sweden’s first follow-up to monitor Sweden’s progress towards the WHO goal of eliminating both hepatitis B and C as a public health threat until 2030.

In 2015, the baseline year according to the definition by the WHO, the estimated prevalence and the notification rates of diagnosed HBV and HCV infections were already low when compared to the rest of the EU [3].

The estimated prevalence of diagnosed chronic HBV infections increased slightly between 2015 and 2018. Since there is no effective curative treatment chronic infections will accumulate as long as the number of new cases exceeds deaths among the infected population. The notification rate of HBV infections on the other hand, decreased by 40% between 2015 and 2018. This decrease was especially striking among men. The majority of notified HBV infections were in immigrants who were likely infected as children in high-prevalence countries and then diagnosed after migrating to Sweden. This steep decrease after 2015 can be explained by reduced immigration to Sweden. In 2015, 162,877 asylum seekers came to Sweden, more than twice as many as in 2014 [22]. Migrants at that time were mostly from Syria and Afghanistan with a predominance of young men [23]. The prevalence of HBV infection in migrants from Syria has been estimated at around 6%, and at 10% among those coming from Afghanistan [24]. In 2018, on the other hand, there were only 21,502 new asylum seekers in Sweden [25]. The notification rate of domestically acquired HBV infections, while already very low, also decreased between 2015 and 2018. Since 2016, all Swedish children have been offered vaccination for HBV, leading to a sharp increase in vaccination coverage. This will likely further reduce the notification rate of domestically acquired infections in the future [10].

The estimated prevalence of diagnosed HCV infections decreased between 2015 and 2018, this is likely due to wider availability of DAA treatment. The rate of notification of newly diagnosed infections also decreased by 20%, which may be a result of several factors that have both reduced the prevalence of injecting drug use and made it safer. Such factors include large awareness campaigns for the risk of blood borne infections after HIV was discovered in the 1980s and HCV in the 1990s. They also include, more recently, increased coverage of needle-exchange programs where both the supply of clean equipment and screening for HCV have possibly lead to a reduced prevalence that in turn leads to a lower risk of being infected. The decrease in the rate of notification can also reflect changes in the prevalence of injecting drug use. The prevalence and notification rate of newly diagnosed HCV infections was highest in men and in young adults, which probably mirrors the epidemiology of injection drug use in Sweden [26], indicating a need to further strengthen needle exchange and other harm-reduction programs. In contrast to HBV, most HCV infections were acquired in Sweden.

We did not observe any change in HBV-related morbidity at the time of diagnosis between 2015 and 2018. The HCV-related morbidity at the time of diagnosis decreased substantially between 2015 and 2018, hopefully indicating shorter time from primary infection to diagnosis. A possible explanation for this is that most of the birth cohort infected in the 1970s and 80 s (before the discovery of HCV) was diagnosed after a long time with HCV infection thus having an increased risk of developing liver-associated morbidity, and we now see a shift towards the diagnosis of HCV infection at a younger age before serious liver damage develops, possibly through the harm reduction programs that offer regular screening. Most deaths among HBV and HCV-infected individuals occurred in older age groups. There was no change in the proportion of deaths attributable to HBV and HCV infections between 2015 and 2018. This was likely due to the short monitoring timespan before the long-term effect of the new DAA treatment will be seen.

These results need to be interpreted in light of some limitations. We focused our analysis on permanent Swedish personal identification numbers, thus excluding temporary Swedish personal identification numbers. This potentially underestimated the true prevalence and notification rates. However, including them would have led to an overestimate as we would not have been able to remove duplicate notifications. We assumed that for persons residing in Sweden for a longer time, a new HBV or HCV notification was created when the person visited health care after obtaining their permanent personal identification number.

We made several assumptions regarding spontaneous clearance and cure rates in response to treatment. Firstly, we assumed the percentage of spontaneous clearance after diagnosis for acute HBV infections to be 5% for infants and 95% otherwise. We acknowledge that taking a sharp cut-off of 1 year of age for spontaneous clearance is likely to be clinically untrue. Since HBV infection among children in Sweden is very rare (mother-to-child transmission in Sweden is < 0.1%), and as a result an extremely small proportion of reported cases are diagnosed for hepatitis B when they are children, changing the cut-off from 1 to 5 years of age or assuming a slower over-time decrease would likely not affect the estimates measured in this paper [27]. Secondly, we assumed no cure rates after any antiviral treatment for treated HBV-infected individuals. People with HBV who are treated with interferon or other antivirals may in reality have achieved functional cure. However, even patients with functional cure from chronic HBV infection still have cccDNA and may be at risk for HCC, leading to this assumption of no cure rates for the presented analyses.

The ICD-10 codes that we used for liver disease were the ones specified in the WHO framework. Even though this gives us the ability to compare our results to other countries using the WHO framework, the codes may not be the most appropriate for a Swedish setting, as demonstrated by a previous study by Duberg et al. [28], and this might have led to an underestimation of the hepatitis associated morbidity and mortality. In addition, the sensitivity and specificity of these codes vary, and we cannot exclude other comorbidities such as alcohol use as causes of these diagnoses. Furthermore, the Prescribed Drug Register was only established in 2005, thus we do not know about treatments before 2005, and this might have led to an overestimation of the prevalence. We assumed that there were no reinfections for hepatitis C in order to simplify our analysis, which might have led to an underestimation of the prevalence and of the notification rates [29].

Finally, our estimates are based on diagnosed and notified cases without estimating the infected but undiagnosed population. The ECDC estimates that only 20%–30% of all HBV and HCV infections are diagnosed in Europe [3]. Previous modelling studies estimated that around 80% of HCV infections in Sweden may be diagnosed [8]. Consequently, prevalence and rates of diagnosed cases do not reflect the true prevalence and incidence of HBV or HCV infections.

## Conclusions

In conclusion, this plan to monitor Sweden’s progress towards the elimination of HBV and HCV as a public health threat by 2030 can be used for regular follow-up. This first monitoring shows encouraging results for the first three years of 2015–2018, though subsequent follow-ups are warranted. Pandemic with COVID-19 has since 2020 possibly affected this elimination work and progress, and future studies are needed to assess the impact of this. We recommend that the progress should be monitored regularly until 2030.

## Data Availability

The datasets generated and/or analysed during the current study are not publicly available due to the data coming from different national governmental organizations in Sweden. Datasets are available from the corresponding author on reasonable request. The corresponding author will check with all organizations involved before sharing the data.

## Abbreviations

ATC: Anatomical Therapeutic Chemical Classification System
CI: Confidence interval
COVID-19: Coronavirus disease 19
DAA: Direct-acting antiviral
ECDC: European center for disease control
HBV: Hepatitis B virus
HCC: Hepatocellular carcinoma
HCV: Hepatitis C virus
ICD-10: 10Th International Classification of Diseases
IFN: Interferon
IgG: Immunoglobulin G
IQR: Interquartile range
WHO: World Health Organization

## References

[1] WHO—Global health sector strategy on viral hepatitis 2016–2021. Towards ending viral hepatitis. 2016

[2] WHO - Monitoring and Evaluation for Viral Hepatitis B and C: recommended indicators and framework. Technical report. 2016.

[3] European Centre for Disease Prevention and Control. Monitoring the responses to hepatitis B and C epidemics in EU/EEA Member States, 2019. Stockholm: ECDC; 2020. https://www.ecdc.europa.eu/sites/default/files/documents/hepatitis-B-C-monitoring-responses-hepatitis-B-C-epidemics-EU-EEA-Member-States-2019.pdf Accessed 21 Jul 2020.

[4] Folkhalsomyndigheten. Statistics.Hepatitis B. https://www.folkhalsomyndigheten.se/folkhalsorapportering-statistik/statistikdatabaser-och-visualisering/sjukdomsstatistik/hepatit-b/. Accessed 21 Jul 2020.

[5] Folkhalsomyndigheten. Statistics.Hepatitis C. https://www.folkhalsomyndigheten.se/folkhalsorapportering-statistik/statistikdatabaser-och-visualisering/sjukdomsstatistik/hepatit-c/ Accessed 21 Jul 2020.

[6] Schweitzer A, Horn J, Mikolajczyk RT, Krause G, Ott JJ (2015). Estimations of worldwide prevalence of chronic hepatitis B virus infection: a systematic review of data published between 1965 and 2013. Lancet.

[7] Polaris Observatory Collaborators (2018). Global prevalence, treatment, and prevention of hepatitis B virus infection in 2016: a modelling study. Lancet Gastroenterol Hepatol.

[8] Folkhalsomyndigheten. Estimate of the number of people living with hepatitis C infection in Sweden in 2015. https://www.folkhalsomyndigheten.se/contentassets/2c53ba20ad2b40778a54485dedac7298/skattning-antalet-personer-lever-sverige-hepatit-c-infektion-16062-webb.pdf. Accessed 21 Jul 2018.

[9] Duberg AS, Blach S, Falconer K, Kåberg M, Razavi H, Aleman S (2015). The future disease burden of hepatitis C virus infection in Sweden and the impact of different treatment strategies. Scand J Gastroenterol.

[10] Folkhalsomyndigheten. Childhood vaccination 2019 yearly report. https://www.folkhalsomyndigheten.se/contentassets/ac113e02858e442391e329c19965091c/barnvaccinationsprogrammet-sverige-2019.pdf. Accessed 15 Dec 2018.

[11] Westin J, Aleman S, Castedal M, Duberg AS, Eilard A, Fischler B (2020). Management of hepatitis B virus infection, updated Swedish guidelines. Infect Dis.

[12] Kåberg M, Navér G, Hammarberg A, Weiland O (2018). Incidence and spontaneous clearance of hepatitis C virus (HCV) in people who inject drugs at the Stockholm Needle Exchange-Importance for HCV elimination. J Viral Hepat.

[13] Lagging M, Wejstål R, Duberg AS, Aleman S, Weiland O, Westin J, for the Swedish Consensus Group (2018). Treatment of hepatitis C virus infection for adults and children: updated Swedish consensus guidelines 017. Infect Dis.

[14] Folkhalsomyndigheten. Case definitions when reporting according to the Infection Control Act. 2022. https://www.folkhalsomyndigheten.se/publikationer-och-material/publikationsarkiv/f/falldefinitioner-vid-anmalan-enligt-smittskyddslagen/. Accessed 20 Sep 2022.

[15] Mortality register at the Swedish Tax Agency. https://www.skatteverket.se/privat/folkbokforing/attvarafolkbokford/folkbokforingsdatabasen.4.3810a01c150939e893f16fe2.html. Accessed 21 Jul 2020.

[16] National Patient Register at the National Board of Health and Welfare. https://www.socialstyrelsen.se/en/statistics-and-data/registers/register-information/the-national-patient-register/. Accessed 21 Jul 2020.

[17] Swedish Prescribed Drug Register at the National Board of Health and Welfare. https://www.lupop.lu.se/lupop-for-researchers/registers/the-swedish-prescribed-drug-register Accessed 21 Jul 2020.

[18] National Mortality Register at the National Board of Health and Welfare. https://www.socialstyrelsen.se/statistik-och-data/register/alla-register/dodsorsaksregistret/. Accessed 21 Jul 2020.

[19] Statistics Sweden. https://www.statistikdatabasen.scb.se/pxweb/sv/ssd/. Accessed 21 Jul 2020.

[20] Aisyah DN, Shallcross L, Hully AJ, O'Brien A, Hayward A (2018). Assessing hepatitis C spontaneous clearance and understanding associated factors-A systematic review and meta-analysis. J Viral Hepat.

[21] Wong GLH, Gane E, Lok ASF (2022). How to achieve functional cure of HBV: stopping NUCs, adding interferon or new drug development?. J Hepat..

[22] Palumbo E (2011). Pegylated interferon and ribavirin treatment for hepatitis C virus infection. Ther Adv Chronic Dis..

[23] Swedish ministry of statistics. Immigration 2015. https://www.scb.se/hitta-statistik/statistik-efter-amne/befolkning/befolkningens-sammansattning/befolkningsstatistik/pong/statistiknyhet/asylsokande-grund-for-bosattning-utlandsk-bakgrund-medborgarskapsbyten-adoptioner-hushallsstatistik-och-medellivslangder-2015/. Accessed 21 Jul 2020

[24] Kowdley KV, Wang CC, Welch S, Roberts H, Brosgart CL (2012). Prevalence of chronic hepatitis B among foreign-born persons living in the United States by country of origin. Hepatology.

[25] Fores. Information on migrations. https://www.migrationsinfo.se/migrationsinfo-sammanfattar-migrationsaret-2018/. Accessed 21 Jul 2020

[26] EMCDDA. Sweden: Country Drugs Report 2019. https://www.emcdda.europa.eu/countries/drug-reports/2019/sweden_en. Accessed 21 Jul 2020

[27] Trépo C, Chan HL, Lok A (2014). Hepatitis B virus infection. Lancet.

[28] Duberg AS, Törner A, Davidsdóttir L, Aleman S, Blaxhult A, Svensson A (2008). Cause of death in individuals with chronic HBV and/or HCV infection, a nationwide community-based register study. J Viral Hepat.

[29] Hajarizadeh B, Cunningham EB, Valerio H, Martinello M, Law M, Janjua NZ (2020). Hepatitis C reinfection after successful antiviral treatment among people who inject drugs: a meta-analysis. J Hepatol.

